# Chronic seronegative spondyloarthropathy following acute *Mycoplasma pneumoniae* infection in a human leukocyte antigen B27-positive patient: a case report

**DOI:** 10.1186/s13256-020-02479-6

**Published:** 2020-09-17

**Authors:** Georgios Pilianidis, Ariti Tsinari, Dimitrios Pandis, Hara Tsolakidou, Nikolaos Petridis

**Affiliations:** Internal Medicine Department, G. Papanikolaou General Hospital, Thessaloniki, Greece

**Keywords:** *Mycoplasma pneumoniae*, Spondyloarthropathy, Reactive arthritis, Sacroiliitis, HLA-B27 positivity

## Abstract

**Background:**

We report a case of a 30-year-old patient who presented with acute *Mycoplasma pneumoniae* infection that was complicated by reactive arthritis and asymmetric proximal myopathy and progressed to chronic spondyloarthropathy. Reactive arthritis and sacroiliitis are unusual extrapulmonary manifestations of *M. pneumoniae* infection, which is a common condition.

**Case presentation:**

A 30-year-old Greek previously healthy man presented to our emergency department with fever, progressively worsening bilateral lower limb weakness, and asymmetric oligoarthritis. Our diagnosis was based on a positive polymerase chain reaction test for *M. pneumoniae* using blood and cerebrospinal fluid and magnetic resonance imaging findings that suggested sacroiliitis. Our patient was also found to be human leukocyte antigen B27 positive. His infection was successfully treated with a 14-day course of doxycycline; the arthritis was treated with naproxen and corticosteroids. His arthritis, which restricted his mobility, improved progressively, and he was discharged without any neurological symptoms.

**Conclusions:**

In our case, an acute *M. pneumoniae* infection eventually progressed to chronic spondyloarthropathy. In our patient, *M. pneumoniae* infection may represent a random event, or it might be a necessary factor for the development of reactive arthritis, asymmetric proximal myopathy, and sacroiliitis, always in combination with the appropriate genetic background. Extrapulmonary manifestations of *M. pneumoniae* may occur even in the complete absence of respiratory symptoms, and the diagnosis of unusual complications, such as reactive arthritis, requires high clinical suspicion and extensive investigation.

## Introduction

Arthritis is one of the extrapulmonary manifestations of *Mycoplasma pneumoniae* infection. The number of infected patients who develop arthritis is not known. Viral and bacterial antigens have been suggested to be triggers for arthritis in susceptible individuals. Although the human immune system makes it difficult to isolate causal agents, the humoral immune response produces detectable serum antibodies that can be detected. A case–control study by Ramirez *et al.* demonstrated a relationship between *M. pneumoniae* infection and rheumatoid arthritis [1].

*M. pneumoniae* infection is referred to in the literature as a cause of extrapulmonary manifestations, including articular and muscular, for all pediatric ages [2]. It has also been described as a triggering agent of reactive arthritis that then progresses to chronic spondyloarthropathy in children [3].

*Mycoplasma* was a term used to refer to any of the class Mollicutes, which include *Mycoplasma* and *Ureaplasma*. The most commonly known species, *M. pneumoniae*, has been associated with the presence of atypical pneumonia. It has also been correlated with infections in other anatomical sites, such as the skin, central nervous system (CNS), blood, heart, and joints.

We report a case of a 30-year-old man with signs of reactive arthritis after *M. pneumoniae* infection, with rapid improvement after doxycycline, naproxen, and corticosteroid therapy.

## Case report

A 30-year-old Greek man presented to our emergency department with a history of fever up to 38.3 °C, sore throat, swollen ankle joints, and bilateral lower limb weakness. His illness began 20 days prior with multiple episodes of diarrhea for which he was prescribed ciprofloxacin and metronidazole for 5 days by his doctor. After cessation of the diarrhea, he developed arthralgias and progressive lower extremity weakness with persistent low-grade fever.

On physical examination, he was febrile with a body temperature of 38.3 °C, heart rate of 120 beats/minute, and blood pressure of 130/90 mmHg; pulse oximetry showed an oxygen saturation rate of 98% in room air. A physical examination of the respiratory tract, the cardiovascular system, and the abdomen was unremarkable.

Examination of his skin and nails was normal. An eye examination was also normal.

Cranial nerve examination was normal; however, a neurological examination revealed asymmetric proximal muscle weakness of his right upper limb and both lower limbs but no distal muscle weakness. Pain, light touch sensation, and joint position sense were intact. Deep-tendon reflexes of his left biceps, triceps, and brachioradialis were brisker than normal. Deep-tendon reflexes of his right upper limb and both lower limbs were normal. Palmar tendon reflexes were normal. He had difficulty standing due to his proximal muscle weakness, but he was able to walk. A computed tomography (CT) scan of his brain was performed with unremarkable findings, followed by lumbar puncture to exclude CNS infection. Cerebrospinal fluid (CSF) analysis revealed pleocytosis with 15 polymorphonuclear cells per field, glucose 56 mg/dl, lactate dehydrogenase 25 U/l, and albumin 19 mg/l. Electromyography showed the presence of many automatic polyphasic motor units in all the examined muscles (right biceps, right quadriceps, right anterior tibialis), especially in the right quadriceps, favoring a myopathic process related to his current *Mycoplasma* infection. His creatine phosphokinase (CPK) was 42 U/l.

A laboratory investigation showed mild normocytic normochromic anemia with hematocrit (Hct) 39.6% and hemoglobin (Hgb) 13.1 g/dl, polymorphonuclear leukocytosis (10,700/mm^3^), an elevated erythrocyte sedimentation rate (125 mm/hour), and increased levels of C-reactive protein (22.4 mg/l), ferritin (586 ng/ml), and fibrinogen. Blood, urine and fecal cultures were all negative.

During the first days of hospitalization, he remained febrile with deteriorating extremity weakness. A chest and abdominal X-ray as well as a heart ultrasound were performed to investigate his fever, with unremarkable findings. An abdominal ultrasound revealed an enlarged spleen (dimensions, 150 mm × 145.5 mm × 78 mm). A tuberculin skin test was negative. A colonoscopy was performed, and inflammatory bowel disease was excluded.

During the first week of his hospital stay, his asymmetric oligoarthritis became more apparent clinically, particularly involving the second metacarpophalangeal joint of his right hand, his left knee joint, and both ankle joints, further limiting our patient’s mobility.

Cervical, thoracic, and lumbar magnetic resonance imaging (MRI) scans were performed to exclude myelopathy, and MRI of his sacroiliac joints revealed sacroiliitis bilaterally. A more thorough investigation of bacterial infection led to the detection of *M. pneumoniae* DNA in his CSF and blood serum samples by polymerase chain reaction (PCR).

*M. pneumoniae*-related reactive arthritis and axial spondyloarthropathy were provisionally diagnosed.

Doxycycline 200 mg daily and a brief course of naproxen 500 mg three times a day were initiated, followed by methylprednisolone 32 mg daily. Our patient’s mobility rapidly improved, inflammation of the affected joints subsided, and inflammatory markers declined. Further blood tests were negative for rheumatoid factor and anti-citrullinated peptide antibody and positive for the human leukocyte antigen (HLA)-B27 antigen. He was discharged after 14 days of hospitalization.

## Discussion

*M. pneumoniae* is a common respiratory pathogen. Extrapulmonary complications can affect many systems, such as the cardiovascular, skin, digestive, hematological, musculoskeletal, nervous, and urogenital tract systems. These complications can be attributed to three major pathophysiologic mechanisms: (1) a direct type in which the presence of the bacterium at the site of inflammation induces the local production of inflammatory cytokines; (2) an indirect type in which the bacterium triggers the autoimmune response and the production of immune complexes without being present at the site of inflammation; and (3) a third type in which vascular thrombosis or vasculitis induced directly or indirectly by the bacterium plays an important role [4].

The first step to the immunologic process is the ability of mycoplasmas to adhere to a cell via adhesins that are homologous with mammalian structures. This resemblance may cause the production of auto-antibodies. However, it cannot be demonstrated whether mycoplasmas are simply cofactors or whether they cause a secondary infection or occur more often in those with abnormal immune systems who also have arthritis [1].

There have been some cases reporting an association between reactive arthritis and acute *M. pneumoniae* infection, mainly in children [3].

Of special note is the fact that, in such cases, arthritis is usually caused by direct inflammation due to *Mycoplasma* at the joint, commonly manifesting as septic arthritis, with a higher prevalence among immunocompromised patients [5].

In our case, our patient was immunocompetent. He presented with asymmetric proximal myopathy. The neurological examination findings, some of which were difficult to fully explain, prompted us to investigate further with CSF analysis, electromyography, and MRI of his brain and spine. CSF analysis revealed polymorphonuclear pleocytosis even though lymphocytic pleocytosis occurs most frequently in *M. pneumoniae* CNS infections [6]. The hypothesis that *M. pneumoniae* infection was the triggering event is supported by the rapid clinical response after the use of doxycycline. Our patient improved clinically without any muscle weakness and with normal and symmetrical tendon reflexes to his upper and lower limbs. Naproxen and methylprednisolone controlled the association with infection reactive arthritis. The diagnosis was based on the clinical picture, blood and CSF PCR findings, and whole-spine MRI results.

The prevalence of spondyloarthropathy and *HLA-B27* gene positivity are correlated in a given population. The correlation is strongest in ankylosing spondylitis. Reactive arthritis following enteric or urogenital infection has a relationship that varies anywhere from below 50% to 85% [7, 8].

Reactive arthritis is considered to be quite rare. The most commonly described organisms causing reactive arthritis are *Chlamydia*, *Campylobacter*, *Salmonella*, *Shigella*, and *Clostridium difficile* [9, 10]*.*

The major finding that differentiates spondyloarthropathy from rheumatoid arthritis and other polyarthritic conditions is enthesitis or inflammation of the sites where the tendons or ligaments insert into the bone. Spondyloarthropathy has a prevalence of 0.03% to 0.04% globally [11]. The major gene involved in its pathogenesis is *HLA-B27*. Many other non-HLA genes have also been implicated in disease pathogenesis. Exposure of the immune system to microorganisms is important for spondyloarthropathy pathogenesis, and several cytokines play a key role in the inflammatory process, including tumor necrosis factor (TNF). Therefore, TNF is a therapeutic target for a class of drugs used to treat spondyloarthropathy [11]. Minor stress trauma triggers inflammation of the enthesis [12]. The chronic inflammatory process causes changes in bone and new bone formation, which is seen in ankylosing spondylitis but not in rheumatoid arthritis.

Peripheral arthritis is characterized by morning stiffness lasting for more than 1 hour, which improves with activity and worsens with rest. Nonsteroidal anti-inflammatory drugs (NSAIDs) offer symptom relief. Patients with peripheral spondyloarthropathy have peripheral arthritis (acute onset, usually the knees and ankles), enthesitis, and dactylitis. Peripheral arthritis is usually asymmetric, as occurred in our patient. Enthesitis is specific to spondyloarthropathy and is usually seen at the insertion of the calcaneal tendon. This causes walking difficulty and tenderness when the tendon is palpated. Dactylitis is found in both psoriatic arthritis and reactive arthritis. Extra-articular symptoms that may coexist are uveitis, iritis, and conjunctivitis, preceding or current gastrointestinal or genitourinary infection, signs of psoriasis or inflammatory bowel disease. A family history of spondyloarthropathy or other autoimmune diseases should also be assessed.

A patient with typical symptoms of peripheral spondyloarthropathy may or may not have predisposing conditions such as psoriasis, inflammatory bowel disease, or preceding infection. If there is a predisposing condition, a diagnosis of psoriatic arthropathy, inflammatory bowel disease-related spondyloarthropathy, or reactive arthritis can be made. If no such relationship is found, then the inflammatory markers and autoimmune profile of the patient, including HLA-B27, as well as imaging studies of the affected spine and peripheral joints should be evaluated.

The initial treatment is a NSAID, sometimes requiring maximum doses to control the symptoms [13]. Glucocorticoids have a limited role in spondyloarthropathy. If symptoms are not controlled by NSAIDs or when the drugs are contraindicated, the next step in treatment depends on whether the disease is axial or peripheral disease. Non-biologic disease-modifying anti-rheumatic drugs (DMARDs), such as methotrexate and sulfasalazine, are not effective in axial disease but are a second-line treatment for peripheral disease [13]. If DMARDs are ineffective or if they are contraindicated or not tolerated, TNF inhibitors are indicated. If the disease is resistant to TNF, a combination with DMARDs should be prescribed.

Reactive arthritis is usually self-limited. It can progress to chronic disease and is treated as any spondyloarthropathy [13].

Our patient has been followed up in our clinic, and his condition has progressed to chronic spondyloarthropathy. Currently, he is being successfully treated with a TNF inhibitor (adalimumab), with satisfactory control of his symptoms and normalization of his inflammatory markers.

In any case of *Mycoplasma*-associated arthritis following an acute *M. pneumoniae* infection, symptoms usually abate with appropriate treatment after 1 month and in some cases 1 year at most.

## Conclusion

Early diagnosis and treatment of *Mycoplasma* infection is important, especially in patients with severe comorbidities. Much work is required to develop an accurate, rapid, and widely available diagnostic test for *Mycoplasma* infections [4]. If a *Mycoplasma* infection is verified, the severity and site of extrapulmonary manifestations should be assessed along with the immunologic status of the patient.

Seronegative spondyloarthropathy is difficult to diagnose and treat, but high clinical suspicion should accompany an acute *Mycoplasma* infection complicated by arthritis.

## Data Availability

Data sharing is not applicable to this article, as no new data were created or analyzed in this case report.
